# Silencing of Renal DNaseI in Murine Lupus Nephritis Imposes Exposure of Large Chromatin Fragments and Activation of Toll Like Receptors and the Clec4e

**DOI:** 10.1371/journal.pone.0034080

**Published:** 2012-03-30

**Authors:** Dhivya Thiyagarajan, Silje Fismen, Natalya Seredkina, Søren Jacobsen, Thomas Elung-Jensen, Anne-Lise Kamper, Christopher Graham Fenton, Ole Petter Rekvig, Elin Synnøve Mortensen

**Affiliations:** 1 Molecular Pathology Research Group, Faculty of Medicine, University of Tromsø, Tromsø, Norway; 2 Department of Rheumatology, Copenhagen University Hospital, Copenhagen, Denmark; 3 Department of Nephrology, Copenhagen University Hospital, Copenhagen, Denmark; 4 The Microarray Platform, Faculty of Medicine, University of Tromsø, Tromsø, Norway; Massachusetts General Hospital and Harvard University, United States of America

## Abstract

Recent studies demonstrate that transformation of mild lupus nephritis into end-stage disease is imposed by silencing of renal DNaseI gene expression in (NZBxNZW)F1 mice. Down-regulation of DNaseI results in reduced chromatin fragmentation, and in deposition of extracellular chromatin-IgG complexes in glomerular basement membranes in individuals that produce IgG anti-chromatin antibodies. The main focus of the present study is to describe the biological consequences of renal DNaseI shut-down and reduced chromatin fragmentation with a particular focus on whether exposed large chromatin fragments activate Toll like receptors and the necrosis-related Clec4e receptor in murine and human lupus nephritis. Furthermore, analyses where performed to determine if matrix metalloproteases are up-regulated as a consequence of chromatin-mediated Toll like receptors/Clec4e stimulation. Mouse and human mRNA expression levels of DNaseI, Toll like receptors 7–9, Clec4e, pro-inflammatory cytokines and MMP2/MMP9 were determined and compared with in situ protein expression profiles and clinical data. We demonstrate that exposure of chromatin significantly up-regulate Toll like receptors and Clec4e in mice, and also but less pronounced in patients with lupus nephritis treated with immunosuppresants. In conclusion, silencing of renal DNaseI gene expression initiates a cascade of inflammatory signals leading to progression of both murine and human lupus nephritis. Principal component analyses biplot of data from murine and human lupus nephrits demonstrate the importance of DNaseI gene shut down for progression of the organ disease.

## Introduction

Lupus nephritis is a serious manifestation of Systemic lupus erythematosus (SLE) and a major predictor of poor outcome [1], [2]. The predominance of chromatin-associated autoantigens involved in lupus nephritis points at deficiencies in the processing and clearance of chromatin from dead cells as central factors in the pathogenesis of SLE [3]–[8]. Enzymatic DNA fragmentation by different endonucleases is significant during apoptotic cell death (reviewed in [9], [10]), and in the elimination of chromatin released from necrotic cells (reviewed in [9]–[11]). In renal tissue, DNaseI represents the major endonuclease [12]. A reduced fragmentation of chromatin during development of nephritis in (NZBxNZW)F1 (BW) mice was shown to coincide with an acquired loss of renal DNaseI mRNA and enzyme activity [4], [5], [13]. This appears when mild mesangial lupus nephritis transforms into end-stage organ disease [5]. Without adequate degradation by DNaseI, chromatin may transform into secondary necrotic chromatin released from apoptotic blebs [7], [8], [14], [15]. In this situation, chromatin fragments may exert central roles in the pathogenesis of SLE. Chromatin may activate the innate immune system through interaction with Toll-like receptors (TLR) 7–9 and the nucleosome-specific adaptive immune system [16]–[19]. Next, exposed chromatin may act as *in situ* target structures for the induced anti-dsDNA antibodies (reviewed in [3]).

The chromatin-mediated stimulation of TLR may also up-regulate certain matrix metalloproteases (MMPs) [20], [21]. For example, engagement of TLRs can up-regulate pro-inflammatory cytokines (TNFα, IFNγ) [22], and Interleukins [23]–[26] by either MAPK, ERK kinase or REL through NFkB gene activation [22], [25]–[28]. Such cytokines can directly up-regulate MMPs [22], [25], [26], [28], [29]. Alternatively, incomplete clearance and degradation of apoptotic cells may transform them into secondary necrotic cell debris [5], [7], [30]–[32]. Necrotic cell debris contains SAP130, which serves as a ligand for the inflammation-related receptor Clec4e [33]. Downstream signalling induced by SAP130-Clec4e interaction also promotes production of pro-inflammatory cytokines [34], [35] and up-regulation of MMPs. Thus, the mechanisms that lead to inflammation in lupus nephritis may therefore involve TLR [16], [36] *and* the Clec4e receptor [37]. Therefore, it is relevant to include the Clec4e receptor in the present study, since we hypothesized that loss of renal DNaseI would result in necrotic transformation of apoptotic cells, with a consequent release of large chromatin fragments and SAP130 [37], [38]. Several studies suggest that TLR signalling is important in the pathogenesis of lupus nephritis [28], [39]–[42], while the role of Clec4e in this context is undetermined.

Secreted MMPs have the potential to disintegrate glomerular basement membranes (GBM) and the mesangial matrix by enzymatic degradation [43]. This biological event may facilitate deposition of chromatin fragment-IgG complexes. MMP2/MMP9 activities are reported to be increased within glomeruli of nephritic, but not pre-nephritic BW mice [5], [13], [44]. Reduced expression of renal DNaseI and increased expression of renal MMPs via the TLR system make a reasonable explanation as to how large chromatin fragments are generated within the kidneys, and how they reach access to membranes and matrices.

As a result, we demonstrate that TLR7–9 as well as the Clec4e receptor and downstream signalling molecules are up-regulated in untreated lupus prone mice, and most importantly, while less pronounced, also in human lupus nephritis ISN/RPS class IV, even though these patients were under treatment. These events promote up-regulation of renal MMP2 and MMP9, with their detrimental effects on glomerular matrices and membranes [28], [44], [45]. From the present data, we conclude that reduced renal chromatin metabolism imposed by DNaseI gene silencing is a key factor to understand how necrotic chromatin-mediated activation of TLR and Clec4e result in a consequent up-regulation of pro-inflammatory cytokines, and increased expression of MMP2 and MMP9. These processes will in the end result in deposition of large chromatin fragments in complex with IgG in GBM, and in fulminant lupus nephritis [5].

## Materials and Methods

### Ethic statements

The National Animal Research Authority (NARA) approved this study (approval ID: 07/11167, ID-178). Coherent analyses on renal biopsies, taken from patients with lupus nephritis, were approved by The Scientific Ethical Committee, Copenhagen ((KF) 01-2006-7214). Informed written consent was given by the patients.

### Murine and human renal tissue samples

Renal tissue was collected from female BW mice (Jackson Laboratory, Bar Harbor, Maine, USA) sacrificed approximately every second weeks (n = 3) from the age of 4 weeks until development of end-stage disease in the BW mice, clinically defined when severe proteinuria developed (≥20 g/L). Tissue was either snapfrozen for protein extraction, or preserved in RNAlaterTM (Qiagen Inc, Valencia, CA, USA) for quantitative mRNA analyses, or embedded in Tissue Tech OCT compound for immunofluorescence analyses. Serum samples were collected at 2–3 week intervals and stored at −80°C. Complete sets of murine data are generated in kidneys from pre-nephritic, antibody negative (Group 1) BW mice (n = 6), BW mice with mesangial nephritis (Group 2, n = 12), and in BW mice with end-stage membrano-proliferative nephritis (Group 3, n = 5). Baseline data on these mice have been published recently [5]. Renal biopsies were taken from patients with lupus nephritis. Entry criteria for the patients were fulfilment of the ACR classification criteria for SLE [46] and clinical indication for renal biopsy. Control samples from morphologically normal cortical tissue were sampled from nephrectomy specimens immediately after the surgical procedure. Paraffin-embedded tissues from non-lupus renal diseases were included.

### RNA isolation and cDNA synthesis

RNA was isolated from RNAlaterTM preserved human kidney tissues using TRIzol (Invitrogen, CA,USA) as described by manufacturer. RNA from murine samples or from human renal proximal tubule epithelial cells (RPTEC) was isolated from RNAlaterTM-preserved kidneys using EZ-1 RNA tissue mini kit (Qiagen, Nordic, Norway) and Qiagen Bio Robot EZ1. The samples were reverse transcribed with random primers using High Capacity cDNA Reverse Transcription kit (Applied Biosystems, Foster City, CA, USA).

### TLR signalling Array Assay (n = 9 mice) and individual quantitative PCR analyses (n = 23 mice)

Quantitative real time PCR (qPCR) was performed using ABI Prism 7900HT Sequence Detection System (Applied Biosystems). RT2 ProfilerTM PCR Array mouse TLR signalling Pathway array plate (PAMM-018) and the accessory master mix were purchased from SABiosciences (Frederick, MD, USA). The cDNA of the respective samples were normalized to a concentration of 1 µg/µl and mixed with the mastermix and used for qPCR. Relative expression levels were calculated using ddCT method. Selected sets of data from these array analyses are given in Table 1, while complete sets of array data are given in Table S1.

**Table 1:** TLR-related signalling: Genes that deviate from levels in kidneys from Group1 BW mice&.

		Mouse Lupus nephritis Group		
Functional molecules	Affected genes	1	2	3
Receptors	Tlr7	1,8 (±1,6)	3,9 (±5,7)	9,6 (±2,9)
	Tlr8	1,5 (±1,2)	6,6 (±5,2)	29,2 (±17,7)*
	Tlr9	1,1 (±0,4)	2,5 (±1,7)	6,2 (±2,2)*
	Clec4e	1,2 (±0,8)	12,9 (±16,6)	33,7 (±25,3)
Co-stimulatory molecules	Cd80	1,2 (±0,8)	7,1 (±4,2)	8,3 (±6,6)
	CD86	1,1 (±0,5)	3,6 (±0,2)	5,6 (±1,6)**
Signalling molecules				
	Muc13	1,4 (±1,3)	1,8(±1,5)	16,7(±25,8)
	Ly86	1,6 (±1,5)	1,7 (±1,0)	11,0 (±1,9)***
	Nfkb2	1,0 (±0,3)	1,7 (±0,4)	2,7 (±0,9)*
	Rel	1,1 (±0,5)	2,6 (±0,1)	3,1 (±1,3)*
	Myd88	1,1 (±0,6)	1,2 (±0,2)	2,4 (±0,4)
Cytokines/Interleukins				
	IFNγ	1,0 (±0,3)	6,2 (±3,6)	6,1 (±5,6)
	TNFα	1,1 (±0,4)	4,0 (±1,0)	12,4 (±10,5)
	Il6	1,1 (±0,7)	6,8 (±8,0)	38,5 (±13,6)**
	IL-10	1,1 (±0,5)	3,9 (±2,2)	9,5 (±5,4)

& Data are given as fold change (± SD) compared with gene expression in pre-nephritic mice.

Pre-designed FAM-labeled gene expression assays (Applied Biosystems) were purchased, with the following accession numbers for individual murine and humane primers and probes: DNaseI Mm01342389_g1, Hs00173736_m1; MMP2 Mm00439506_m1, Hs00234422_m1; MMP9 Mm00442991_m1, Hs00234579_m1; TNF α Mm00443258_m1, Hs 00174128_m1; INFγ Mm00443258_m1, Hs 00174128_m1; INFα1 Hs04189288_g1; Clec4e Mm00490873_m1, Hs00372017_m1; TLR7 Mm00446590_m1 Hs 00152971_mH; TLR8 Mm04209873_m1 Hs 00607866_m1; TLR9 Mm00446193_m1, Hs 00928321_m1; β-Actin 4352933E, 4333762F: Myd88 Hs 00182082_m1; IL-6 Mm01210732_g1, Hs 00985639_m1; IL-10 Mm99999062_m1, Hs 00961622_m1; TBP 4333761F, RPLPO Mm00446973_m1. RPLPO (large ribosomal protein) was used as endogenous control for human samples and β-Actin and Tata Box binding protein (TBP) for murine samples. The relative expression levels were calculated using the ddCT method relative to pre-nephritic kidneys (for mice) and normal renal tissue (for human analyses).

### Laser capture micro-dissection of murine kidneys

Ten micrometer thick kidney cryosections were prepared and immediately fixed in zinc buffer (40 mM ZnCl_2_, 30 mM ZnAc_2_ and 600 mM CaAc_2_ in 0.1 M Tris, pH 7.4) for 5 min, stained with hematoxylin, dehydrated, air-dried, and overlaid with liquid coverglass (PALMZeiss, Bernreid, Germany). Cortex was collected by laser capture micro-dissection on a PALM Laser-MicroBeam System. For each sample, tissue from 10 sections were collected, lysed in TRIzol (Invitrogen, Carlsbad, CA, USA), and stored at −70°C for further RNA isolation.

### Antibodies

Rabbit IgG antibodies specific for mouse and human MMP2 (ab52756), MMP9 (ab73734), TLR7 (ab59921), TLR8 (ab53630), TLR9 (ab53596) were all from Abcam (Cambridge, UK), while antibodies against DNase I (sc30058) and Clec4e (SC-161489) were from Santa Cruz (Santa Cruz Biotechnology, Inc, Santa Cruz, CA, USA), and antibodies against β-actin (A2066) were from Sigma-Aldrich (St Louis, MO, USA). Alexa 488-conjugated F(ab)_2_ anti-rabbit IgG antibodies were from Invitrogen Life Technologies (Carlsbad, CA, USA).

### Immunofluorescence and immunohistochemistry analyses

Four micrometer thick sections of OCT-embedded kidneys were blocked for 30 min in 1% BSA in phosphate-buffered saline (PBS) followed by washing and 30 min incubation with rabbit anti-DNaseI, rabbit anti-mouse/human TLR7–9 and goat anti-Clec4e antibodies. Slides were washed and incubated further for 30 min with Alexa 488-conjugated F(ab)_2_ anti-rabbit IgG antibodies. Normal goat and rabbit IgG were used as negative controls. The sections were analysed by an Olympus BX51 microscope. Immunohistochemical staining was performed as described [4] and Polink-2 Plus HRP with DAB kit (Newmarket Scientific, UK) used as detection system.

### Cell culture experiments

Human renal proximal tubule epithelial cells (RPTEC) were purchased from Clontec (Lonza, Switzerland). The cells were grown in Clontec REGM™ BulletKit (CC-3190) containing Renal Epithelial Cell Basal Medium with the following growth supplements: hEGF, Hydrocortisone, Epinephrine, Insulin, Triiodothyronine, Transferrin, GA-1000, and fetal bovine serum at 37°C in 95% humidified air and 5% CO_2_. The cells were grown to 80% confluence and were activated with TNFα (Sigma-Aldrich, St. Louis, MO, USA), 0, 15, 30, or 60 ng/ml, or with IFNγ (Sigma-Aldrich), 30, 60, 90, 120 or 240 ng/ml, and the cells were harvested at 3–6 h for TNFα, and 3–24 h for IFNγ, and cellular expression of MMP2 and MMP9 was analysed by qPCR.

### DNaseI Gel Zymography

DNaseI gel zymography was performed exactly as described [5] using proteins extracted from snap frozen kidney sections from BW mice.

### Western blot

Proteins were extracted from homogenized snap frozen kidneys (mouse) or frtom the protein phase from the Trizol procedure (human samples). The protein concentration was determined with standard BCA assay (ThermoScientific, Oslo, Norway). The protein concentration was normalized to 0,2 µg/µl and 10 µl were loaded into Nu PAGE gel (Invitrogen). Electrophoresis and western blotting were performed according to standard procedures given by Invitrogen. Membranes were blocked with 5% (w/v) skimmed milk for 1 h before application of primary antibodies specific for MMP2, MMP9 and DNaseI overnight at 4°C. Binding was revealed by chemiluminescence detection. Determination of molecular weight was done by comparison with MagicMark XP molecular weight markers (Invitrogen).

### ELISA

ELISA kit for mouse MMP9 was obtained from R&D Systems (Abinodon, UK) and mouse MMP2 from RayBiotech, Inc. (Norcross GA 30092, USA). The analyses were done according to the instructions provided by the manufacturers and optical density was measured spectrophotometrically.

### Statistics

Data are presented as mean (±SD). An unpaired t-test was used to calculate differences of qPCR results in this study excepting array date where a one-way ANOVA with Dunett post hoc test was performed; p<0.05 was considered significant. The rcor.test function from the R language ltm package was used to generate correlations and significances presented in the square matrix Table 2, in which all observations were included. Spearman was used for correlation testing. A principal component analysis (PCA) was performed on the same set of data and a biplot drawn with the R biplot function. The PCA biplot is aimed to optimally display variances and not correlations. In the PCA biplot the direction, and length of the variable vectors (arrows) give a good indication as to which variable(s) had the largest influence, positive or negative, in discriminating the various samples.

**Table 2:** Correlations and significances.

	Age	DNase.I	EDS.MM*	EDS.GBM*	Anti-DNA.titer	TLR7	TLR8	TLR9	Clec4e	MMP2	MMP9	Proteinuria
Age	*****	0,274	0,344	0,353	0,282	0,486	0,475	0,533	0,448	0,081	−0,053	0,355
DnaseI	0,206	*****	0,396	−0,391	0,081	−0,292	−0,189	−0,256	−0,265	−0,227	−0,318	−0,420
EDS.MM	0,108	0,061	*****	−0,550	0,347	−0,194	−0,225	−0,263	−0,370	−0,163	−0,131	−0,475
EDS.GBM	0,099	0,049	0,006	*****	−0,055	0,710	0,766	0,834	0,862	0,339	0,241	0,947
Anti-DNA.titer	0,192	0,715	0,105	0,802	*****	0,045	0,166	0,393	0,046	0,016	0,347	−0,020
TLR7	0,019	0,176	0,376	<0.001	0,838	*****	0,534	0,759	0,800	−0,075	0,086	0,681
TLR8	0,022	0,388	0,303	<0.001	0,449	0,009	*****	0,840	0,611	0,634	0,058	0,749
TLR9	0,009	0,238	0,226	<0.001	0,064	<0.001	<0.001	*****	0,781	0,302	0,297	0,768
Clec4e	0,032	0,221	0,082	<0.001	0,835	<0.001	0,002	<0.001	*****	0,092	0,235	0,898
MMP2	0,714	0,297	0,458	0,114	0,941	0,734	0,001	0,162	0,676	*****	−0,031	0,324
MMP9	0,812	0,139	0,552	0,267	0,105	0,697	0,794	0,169	0,280	0,888	*****	0,202
Proteinuria	0,096	0,046	0,022	<0.001	0,927	<0.001	<0.001	<0.001	<0.001	0,131	0,355	*****

* EDS MM and EDS GBM: Electron dense structures in mesangial matrix and in glomerulus basement membranes.

## Results

### TLR7–9 and Clec4e and down-stream signalling are activated during progression of murine lupus nephritis

**Figure 1:**
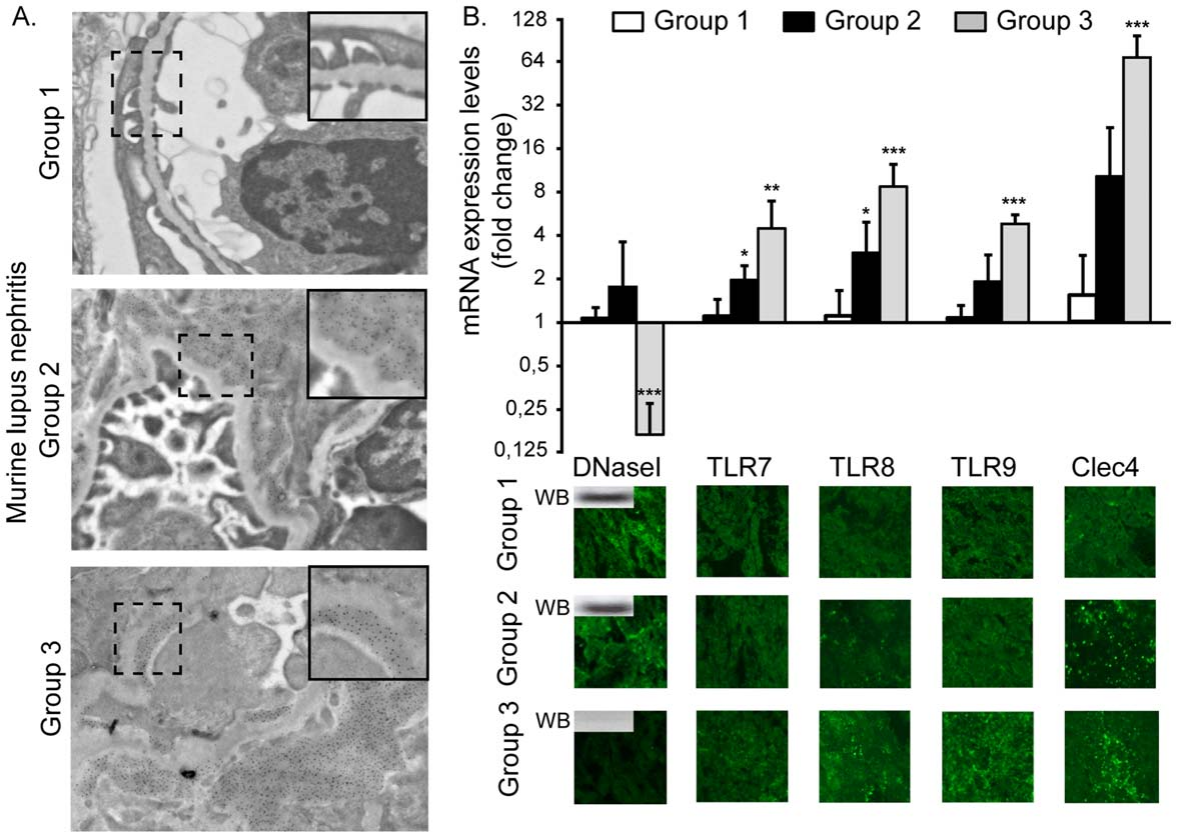
Renal expression of DNaseI, TLR and Clec4e in (NZBxNZW)F1 mice grouped according to glomerular location of EDS. The mice were sorted into 3 main groups according to kidney morphology (A); pre-nephritic mice (Group 1, no EDS (n = 6); mice with mesangial EDS deposits (Group 2 (n = 12)), or mice with EDS in GBM (Group 3 (n = 5)). Magnification ×40 k. In B, mRNA expression levels for renal DNaseI, TLR7–9 and for the Clec4e receptor with corresponding expression of proteins in kidneys of group 1–3 mice are demonstrated. DNaseI gene expression levels correlate inversely with TLR7–9 and Clec4e receptor expression levels, both with respect to transcription and translation of the genes in Group 3 of BW mice. For statistics, see Table 2. Inserts in the DNaseI immunofluorescence panels represent western blots of the DNaseI. An unpaired t-test was performed (*: p<0,05; ***: p<0,0001).

Baseline data for the BW mice included in this study has been published recently [5]. These mice are grouped according to morphology of the kidneys and presence or absence of serum anti-dsDNA antibodies: Group 1 mice have no deposits of immune complexes in the kidneys; Group 2 mice have chromatin-IgG complex deposits, seen as electron dense structures (EDS, as described in both murine and human lupus nephritis [6], [47]) in the mesangium; Group 3 mice have EDS in the mesangium and within the GBM. This separation into 3 groups is in accordance with the analyses published recently [5], [6] (see Figure 1A). Mice from each group (n = 3) were further analysed by a TLR signalling qPCR array assay on genes linked to the TLR and the Clec4e signalling. We observed that genes which are up-regulated at any stage of murine lupus nephritis could be systematically divided into the following subgroups (Table 1): *i.* Receptors (the TLR7–9 and Clec4e); *ii.* Signalling molecules (Myd88, Muc13, Ly86, Nfkb, and Rel); *iii.* Co-stimulatory molecules (CD80/CD86); and *iv.* cytokines and interleukins (TNFα, IFNγ. IL-6, IL-10). Those who are significantly up-regulated are marked by asterisks. The complete TLR signalling results are presented in Table S1.

### DNaseI gene shut-down correlates with activation of TLR7–9 and Clec4e

To further explore DNaseI, TLR and Clec4e gene expression in a larger group of BW mice, individual, selected genes were analysed by qPCR for Group 1 (n = 6), Group 2 (n = 12), and in Group 3 (n = 5) BW mice. In Figure 1B, mean values (±SD) for DNaseI, TLR7–9 and Clec4e mRNA in each mouse group are presented, and significant differences are indicated by asterisks. The data demonstrate that DNaseI gene expression was nearby completely silenced in Group 3 mice, while TLR7–9 and Clec4e mRNA levels increased significantly during disease progression.

In agreement with the qPCR results, renal expression of the DNaseI protein in Group 3 mice was undetectable by immunofluorescence and western blot assays compared to staining intensity in Group1 and Group 2 mice (Figure 1B, western blot results are inserted in panels in Group 1–3 kidneys). Furthermore, in situ expression of the TLR7–9 and the Clec4e (Figure 1B) proteins correlated well with the respective qPCR levels (Figure 1B). Since Clec4e regulation in lupus nephritis is not determined previously, we also analysed if this receptor was selectively up-regulated in nephritic kidneys or in other organs expressing this protein, like the spleen. Although Clec4e gene expression increased immensely in kidneys during progression of the disease from mesangial (Group 2) to membrano-proliferative nephritis (Group 3, Figure 1B), Clec4e expression levels in spleens remained stable at baseline levels throughout the disease process (data not shown).

By micro-dissection, and mRNA analyses of renal cortex and medulla, DNaseI was silenced in both compartments of the kidneys from Group 3 BW mice, thus demonstrating that DNaseI gene silencing is not linked to certain renal structures (data not shown), similar to the DNaseI staining pattern observed by immunofluorescence analyses of the same kidneys (Figure 1B), showing a uniform loss of the DNaseI protein.

### Silencing of renal DNaseI correlates with exposure of chromatin, while exposure of chromatin correlates with TLR7–9 and Clec4e activation

**Figure 2:**
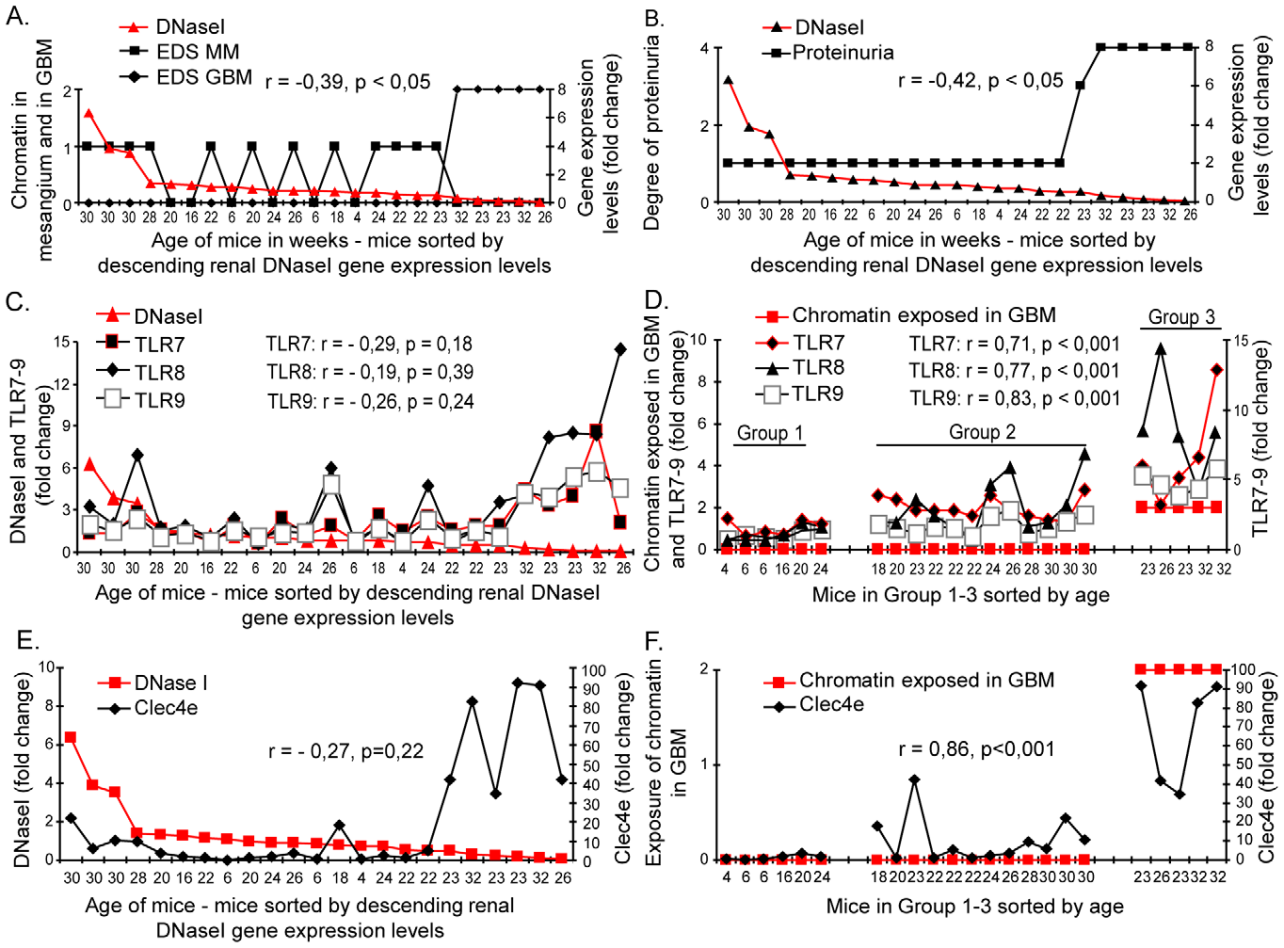
Silencing of renal DNaseI correlates with chromatin deposition in GBM and severe proteinuria, while exposure of chromatin correlates significantly with activation of TLR7–9 and Clec4e. Reduced expression of renal DNaseI correlates inversely with chromatin-IgG deposits in GBM (A) and with severe proteinuria (B). TLR7–9 activation is not significantly correlated with silenced renal DNaseI (C), while a consequence of reduced DNaseI enzyme activity, i.e. exposure of chromatin in e.g. GBM demonstrate a strong and highly significant correlation with TLR7–9 activation (D). ). Similarly, the Clec4e receptor activation is not significantly correlated with silenced renal DNaseI (E), while exposure of secondary necrotic chromatin demonstrate a strong and highly significant positive correlation with Clec4e activation (F).

Deposits of large chromatin fragment-IgG complexes in GBM was observed solely in kidneys of mice from Group 3 with the lowest renal DNaseI gene expression levels (r = −0,39, p<0,05, Figure 2A), and low DNaseI gene expression also correlated inversely with severe proteinuria (r = −0,42, p<0,05, Figure 2B). Renal expression of MMPs is thought to be instrumental in promoting large chromatin-IgG complex deposits in GBM [5], [45], [48]. Since MMPs may be imposed by TLR activation [20], [21], [49], [50], we hypothesized that loss of DNaseI is not directly correlated with TLR/Clec4e gene activation, but rather indirectly through reduced fragmentation and a consequent in situ exposure of secondary necrotic chromatin fragments (for TLRs) and SAP130 (for Clec4e, [33], [51]

In harmony with this assumption, we demonstrate that silencing of the renal DNaseI gene expression did not directly correlate with up-regulation of TLR7–9 gene expression (for correlations and significances, see Figure 2C and Table 2). However, renal exposure of large undigested chromatin fragments correlated strongly and significantly with increased TLR7–9 gene expression (Figure 2D, and Table 2).

Results of analyses of the Clec4e receptor activation strengthen this observation. As loss of DNaseI did not correlate significantly with activation of Clec4e (Figure 2E, Table 2), renal exposure of chromatin fragments correlated highly significantly with increased expression of Clec4e (Figure 2F, Table 2).

### Expression of MMP2 and MMP9 during progressive lupus

TLR activation can directly, or indirectly by increased expression of TNFα and INFγ, up-regulate MMP2 and MMP9 [22], [25], [26], [28], [29]. Both TNFα and INFγ were found to be up-regulated in the murine kidneys during progression of lupus nephritis when analyzing all mice in all groups (data not shown), as previously demonstrated by others [52]–[56]. Thus, the results demonstrated in Figures 1 and 2, therefore, may link TLR and Clec4e stimulation with up-regulation of MMP2 and MMP9, and in cell culture experiments we observed that TNFα and INFγ up-regulated MMP9 and MMP2, respectively, in RPTEC (data not shown).

**Figure 3:**
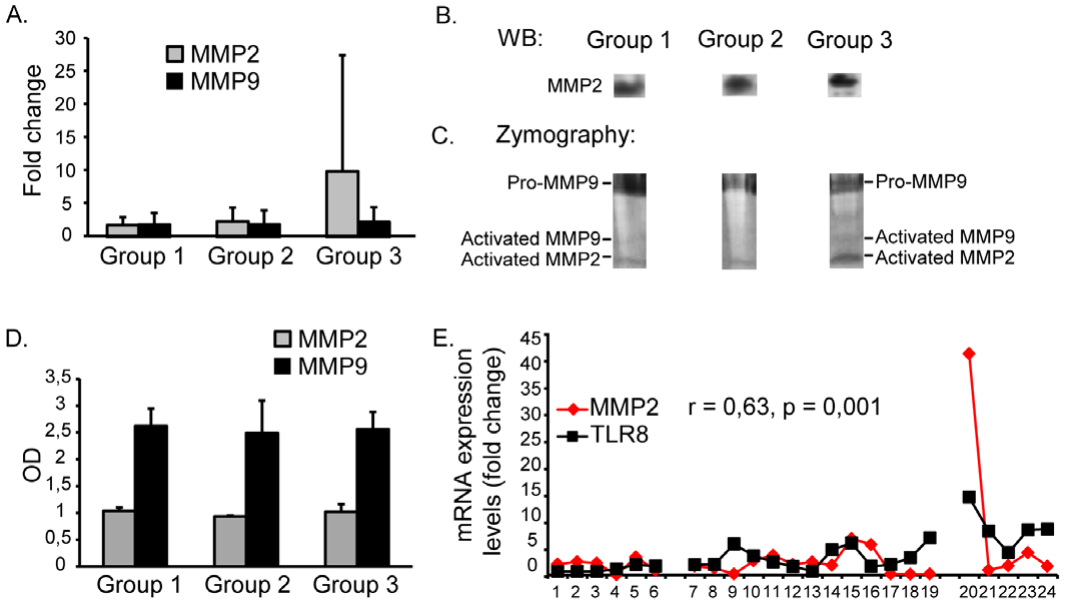
Expression of the MMP2 and MMP9 in kidneys of BW mice. Data demonstrate an insignificant increase in renal MMP2, but not in MMP9 mRNA levels in Group 3 mice compared with mice from Group 1 and 2 (A), and a corresponding increase in MMP2 protein (western blot, B) or in activated MMP2 (zymography, C). Serum concentrations of MMP2 and MMP9 were stable in all stages of the disease as demonstrated by quantitative ELISA for MMP2 and MMP9 (Figure 3D). In agreement with the hypothesis that MMP2 gene expression is linked to activation of TLRs, TLR8 correlates significantly with expression of MMP2 (r = 0,63, p<0,001, E).

Data in Figure 3 demonstrate an insignificant increase in renal MMP2, but not in MMP9 mRNA levels (Figure 3A), and a corresponding increase in MMP2 protein (western blot, Figure 3B) or in activated MMP2 (zymography, Figure 3C) in Group 3 kidneys. On the other hand, serum concentrations of MMP2 and MMP9 were stable in all stages of the disease as demonstrated by quantitative ELISA for MMP2 and MMP9 (Figure 3D). In agreement with the hypothesis that MMP2 gene expression is linked to activation of TLRs, e.g. TLR8 correlates significantly with expression of MMP2 (r = 0,63, p<0,001, Figure 3E).

### Expression patterns of DNaseI, TLR7–9, and Clec4e mRNA and corresponding proteins in kidneys from patients with lupus nephritis

Human samples are divided into 2 groups according to kidney morphology; normal controls (n = 3) and patients with lupus nephritis (ISN/RPS class II (n = 1) and class IV (n = 4) [57]). All ISN/RPS class IV patients were treated with immunosuppressants at the time when biopsies were taken (Table 3).

**Table 3:** Basic clinical, serological and histological data.

Patient ID	Gender/age (years)	ACR criteria^a^	Disease duration	ISN/RPS class lupus nephritis	Activity/Chronicity score	Anti-dsDNA antibody	s-Creatinin (µmol/L)	Proteinuria (g/24 h)	Therapy
LN1	♀/39	1,4,5,7,9,10,11	2 months	IV	6/1	400 (cut-off 15)	78	2.4	Prednisolone H-Chlorochine
LN2	♀/18	5,7,10,11	6 weeks	IV	7/1	>400 (cut-off 15)	68	3.6	Prednisolone
LN3	♂/22	1,4,7,9,10,11	3.5 years	IV	9/10	>400 (cut-off 15)	357	9.0	Prednisolone ***Mycophenolate***
LN4	♀/32	5,7,9,10,11	11 years	IV	6/2	>400 (cut-off 15)	66	1.0	Prednisolone
LN6	♀/40	4,6,7,9,10,11	11 months	I	0/3	153 (cut-off 35)	57	2.0	None

a ACR-criteria: 1 = malar rash, 2 = discoid rash, 3 = photosensitivity, 4 = oral ulcers, 5 = arthritis, 6 = serositis, 7 = renal disorder, 9 = hematologic disorder, 10 = immunologic disorder: Anti-dsDNA, anti-Sm, and/or anti-phospholipid antibodies, 11 = ANA.

**Figure 4:**
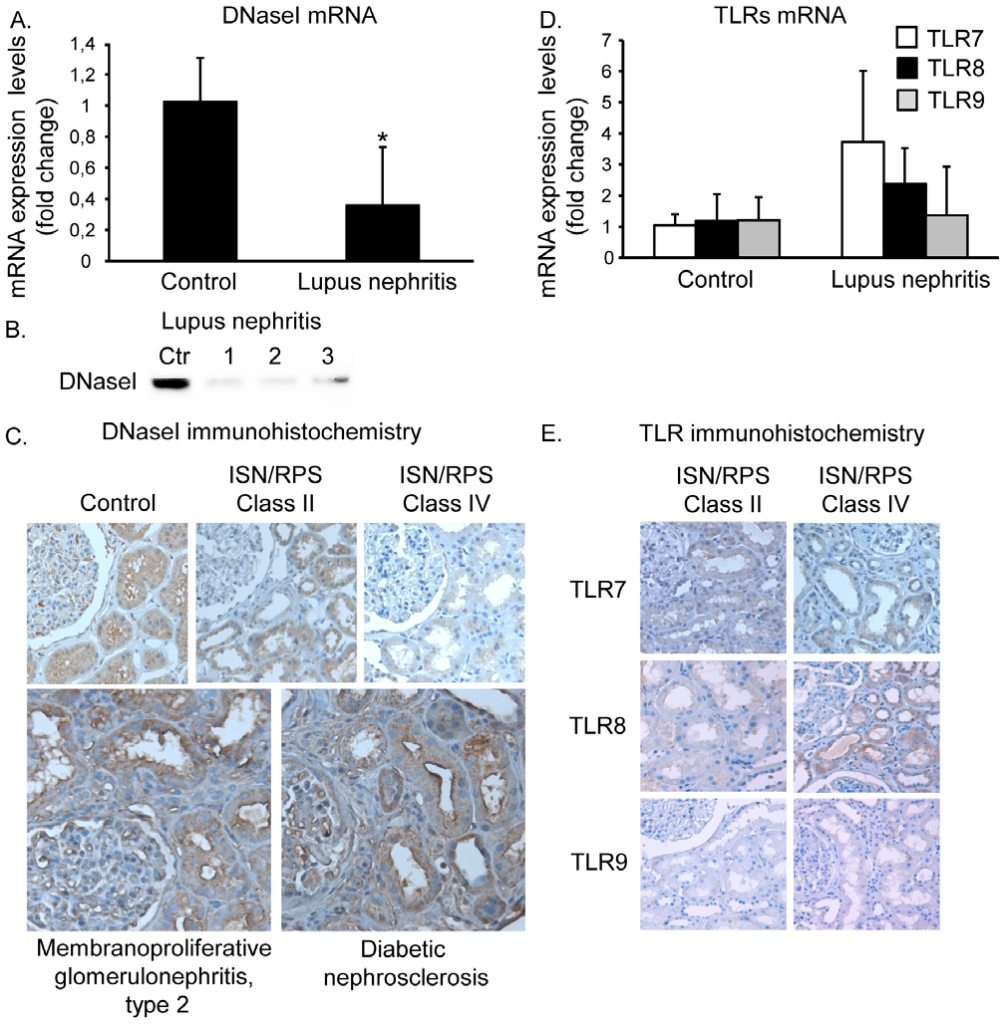
Expression patterns of DNaseI, TLR7–9 in kidneys from patients with lupus nephritis. A profound silencing of DNaseI gene expression in kidneys with membranoproliferative lupus nephritis (ISN/RPS class IV, A) compared with DNaseI mRNA levels in control kidneys. Western blot analyses of renal proteins demonstrated a single band corresponding to the MW of human DNaseI (B). Low band intensities were solely found in the kidney samples that demonstrated considerably reduced DNaseI mRNA levels (LN 1–3, A and B). Similarly, immunohistochemistry analyses of kidney sections demonstrated strong staining intensity of the DNaseI protein in normal control kidneys (C) and in ISN/RPS class II kidneys (C). In ISN/RPS class IV kidneys, DNaseI staining was barely detectable (C), in agreement with the low DNaseI mRNA levels in these patients. Control analyses in other renal diseases (like diabetic nephrosclerosis or membranoproliferative glomerulonephritis type 2) demonstrated DNaseI staining intensity similar to that in normal kidneys, and kidneys with ISN/RPS class II lupus nephritis (C). The qPCR analyses revealed that TLR7 and TLR 8 were up-regulated in class IV nephritis, although without reaching statistical significance (D). The TLR 9 and Clec4e (data not shown) mRNA levels did not differ at all from the control group. In harmony with this, immunohistochemical staining of kidneys with class IV lupus nephritis revealed increased staining intensity of TLR8 (E). An unpaired t-test is performed (*p<0,05).

Similar to results obtained in Group 3 BW mice, we observed a profound silencing of DNaseI gene expression in kidneys with membrano-proliferative lupus nephritis (ISN/RPS class IV, Figure 4A) compared with DNaseI mRNA levels in control kidneys. Very low DNaseI mRNA levels were observed in 3 of the 4, while patients LN4 presented normal DNaseI mRNA level although having class IV nephritis. However, this patient had been on treatment continuously the last 11 years (Table 3), which may have modified the disease process. Western blot analyses of renal proteins demonstrated a single band corresponding to the MW of human DNaseI (Figure 4B). Low band intensities were solely found in the kidney samples that demonstrated considerably reduced DNaseI mRNA levels (LN 1–3, Figure 4A and 4B). Similarly, immunohistochemistry analyses of kidney sections demonstrated strong staining intensity of the DNaseI protein in normal control kidneys (Figure 4C) and in ISN/RPS class II kidneys (Figure 4C). In ISN/RPS class IV kidneys, DNaseI staining of LN 1–3 was barely detectable (Figure 4C), in agreement with the low DNaseI mRNA levels in these patients. Proteins from kidneys of LN4 were not available for this analysis. Control analyses in other renal diseases (like diabetic nephrosclerosis or membrano-proliferative glomerulonephritis type 2) demonstrated DNaseI staining intensity similar to that in normal kidneys, and kidneys with ISN/RPS class II lupus nephritis (Figure 4C). Taken together, shut-down of the renal DNaseI gene expression seems to be an event linked to progression of both murine and human lupus nephritis, as has been indicated in a previous pilot study [13]. Thus, DNaseI shut down in progressive lupus nephritis is not a general phenomenon linked to e.g. renal inflammation.

The qPCR analyses revealed that TLR7 and TLR 8 were up-regulated in class IV nephritis, although without reaching statistical significance (Figure 4D). The TLR 9 and Clec4e mRNA levels did not differ at all from the control group (data not shown). In harmony with this, immunohistochemical staining of kidneys with class IV lupus nephritis revealed increased staining intensity of TLR8 (see Figure 4E for details on each TLR). The discrepancy of TLR expression between murine and human nephritis may be due the fact that these patients had been treated with immunosuppressive drugs while mice were not. The LN6, having mesangial lupus nephritis ISN/RPS class II, had normal levels of DNaseI, TLR7–9 and Clec4e mRNA levels (data not shown), similar to BW mice with mesangial nephritis.

### Expression patterns of MMP2 and MMP9 in kidneys from patients with human ISN/RPS class IV lupus nephritis

**Figure 5:**
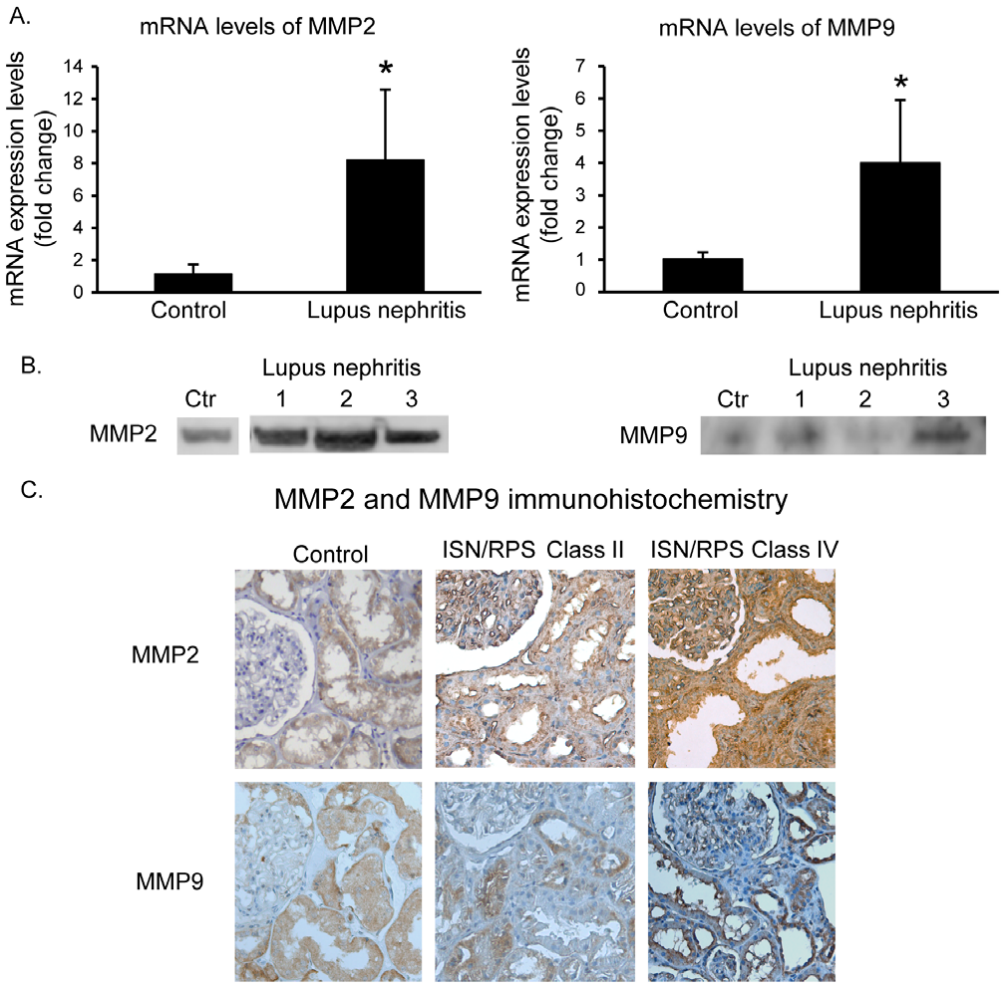
Expression of MMP2 and MMP9 in kidneys from patients with human ISN/RPS class IV lupus nephritis. **T**he MMP2 and MMP9 mRNA levels were significantly up-regulated in class IV lupus nephritis compared with control kidneys (A). The high MMP2 and MMP9 mRNA levels were also reflected by increased protein levels as demonstrated by western blots (B) and by immunohistochemistry (C). Taken together, MMP2 and MMP9 mRNA and protein levels are highly expressed in human class IV lupus nephritis. An unpaired t-test is performed (*p<0,05).

As demonstrated in Figure 5A, the MMP2 and MMP9 mRNA levels were significantly up-regulated in class IV lupus nephritis. The high MMP2 and MMP9 mRNA levels were also reflected by increased protein levels as demonstrated by western blots (Figure 5B) and by immunohistochemistry (Figure 5C), thus demonstrating that these proteins are strongly expressed in class IV kidneys.

### Overall statistical and PCA biplot analyses of the data obtained in this study

**Figure 6:**
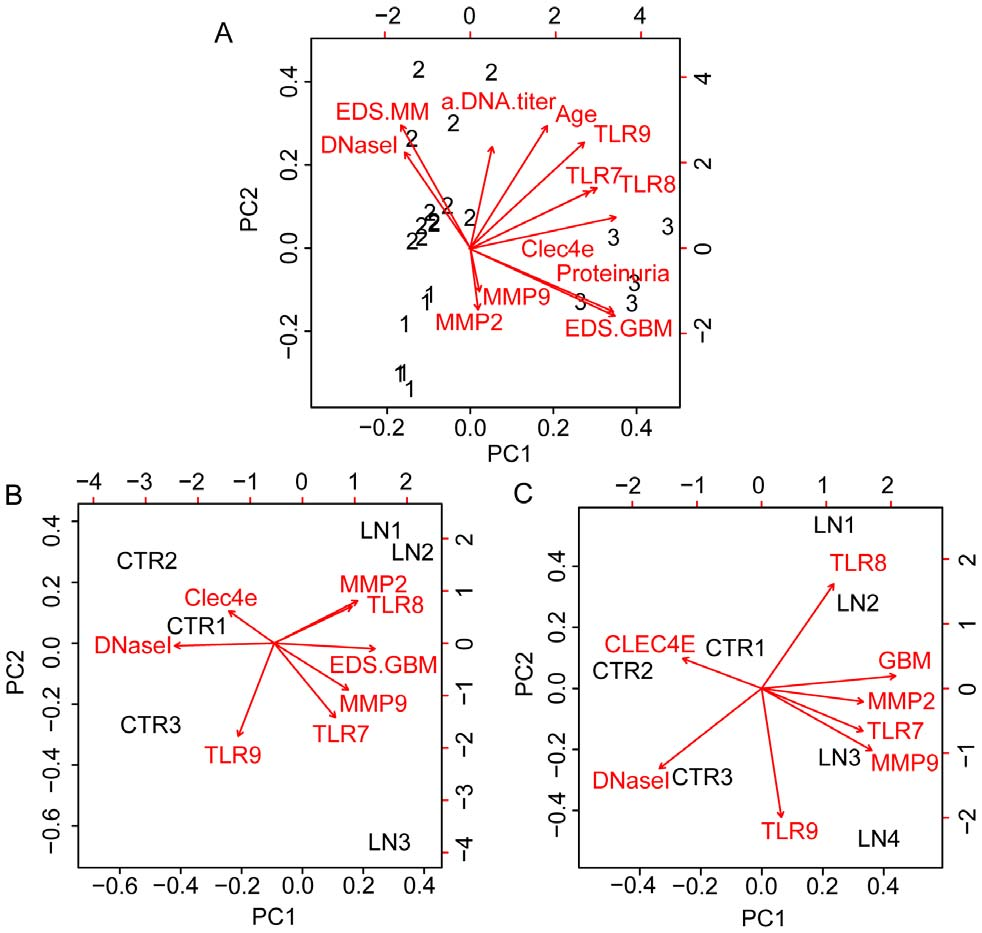
Principal component analysis (PCA) of murine (A) and human (B,C) parameters included in this study. PCA biplots aim to optimally display variances and not correlations. The angles between the various biplot axes serve as good indicators of the correlations among the variables (shown as arrows). Similarly, the position of the samples of individual mice (shown as the signs 1, 2 and 3 for Group 1–3 mice) relative to the arrows, provide good indications as to which variable(s) have had the largest effect on disease progression. The result of the biplots demonstrates that groups emerging from this analysis perfectly correlated with the groups of BW mice as defined in Figure 1, defined as pre-nephritic BW mice (Group 1), BW mice with deposits of EDS in the mesangial matrix (Group 2) or with deposits in the GBM (Group 3). In B, a similar biplot has been generated for the human data, where the 3 patients with low DNaseI expression levels are included. In C, all 4 patients with lupus nephritis class IV are included, i.e. also the patient with normal renal DNaseI. As is demonstrated, the vector for DNaseI differs in B and C, thus demonstrating the impact of DNaseI levels on the biplot. For all biplots, the most striking observations are that DNaseI vector points away from the diseased individuals (to the left in the biplots), while MMP2, MMP9, TLRS and EDS associated with GBM are clustered and points at the most severely diseased murine and human individuals, in harmony with the statistical analyses demonstrated in Table 2.

Table 2 shows a square matrix where the upper diagonal part demonstrates Spearman correlation coefficients. The lower diagonal part presents the corresponding p-values for all murine data included in this study. In Figure 6, the result of a principal component analysis (PCA) biplot drawn with the R biplot function is demonstrated. The PCA biplot is aimed to optimally display variances and not correlations. The angles between the various biplot axes are good indicators of the correlations among the variables (shown as arrows). In A, the murine, and in B the human biplot on LN 1–3, and in C the human biplot for LN 1–4 are presented. In B and C the biplot varies since in B all data are from the 3 kidneys with low DNaseI, while in C, the vectors is shown to differ due to the fact that one patient (LN4) had normal renal DNaseI. The result of the murine PCA biplot (Figure 6A) demonstrate that the mice confine perfectly into three groups, one pre-nephritic (Group 1, all mice labelled with 1), one with mild mesangial nephritis (Group 2, mice labelled with 2), and one with end-stage membrano-proliferative nephritis (Group 3, mice labelled with 3). This result confirms that the parameters used to group BW mice as in Figure 1A are biologically relevant. In sum, the data demonstrate a highly significant inverted correlation between proteinuria/EDS in GBM and DNaseI gene expression, while EDS in GBM was significantly associated with activation of TLR7–9 and clec4e (Table 2).

Similarly, the position of individual human controls (ctr1–3) and patients (LN1–3 (B)) or (LN1–4 (C)) relative to the vectors provides good indications as to which variable(s) have had the largest effect in each individual patient. As is evident in data presented in Figure 6, the DNaseI vector points towards normal murine (Group1) and human (ctr 1–3) individuals due to its inverse impact on disease progression.

The data presented in Table 2 and in Figure 6A–C are relevant to explain biological events imposed by the DNaseI shut-down and its role for progression of lupus nephritis. In sum, the vector that point at chromatin-IgG deposits in GBM are clustered with vectors pointing at TLR7–9, Clec4e, MMP2 and MMP9, indicating an interdependency between them, while the DNaseI vector points the opposite direction due to its negative correlation with advanced lupus nephritis.

## Discussion

At a certain time point in the life of BW mice, the renal DNaseI mRNA and enzyme activity is inevitably lost. Acquired silencing of renal DNaseI gene expression appears to have an immense pathogenic impact on progressive lupus nephritis [4], [5], [13], [58]. The clinical consequence of renal DNaseI gene silencing is an inevitable transformation of mild mesangial lupus nephritis into end-stage organ disease. In this respect, two questions are regarded important to answer; *i.* why the renal DNaseI gene is silenced (studies in progress); and *ii.* if loss of DNaseI activity is the event that imposes increased MMP activity through interaction of un-fragmented secondary necrotic chromatin with TLRs and the Clec4e receptor (present study).

However, exposure of chromatin may not by itself be sufficient to impose clinical lupus nephritis. For example, similar in situ exposure and retention of large un-fragmented chromatin fragments have been described in several experimental nuclease deficiencies on non-autoimmune backgrounds (see e.g. [9], [59]–[61]). The clinical consequence of chromatin fragments in these experimental nuclease deficiencies differ from that in kidneys of individuals with lupus nephritis, simply because in the latter, antibodies to chromatin are present. Thus, antibodies to dsDNA may be the factors that *render* exposed chromatin pathogenic.

In this study we analysed the pathophysiological effect of renal exposure of chromatin fragments. The basic hypothesis was that exposure of chromatin may be the factor that indirectly up-regulate expression of MMPs. This up-regulation may be induced by at least 3 different pathways: i. directly through interaction of chromatin with TLR [20], [21], [49]; ii. indirectly through TLR -mediated up-regulation of TNFα and IFNγ, both having the capacity to induce increased expression of MMP2/MMP9 enzyme activity [23]–[25]; or iii. indirectly through downstream signalling induced by interaction between debris released from apoptotic and secondary necrotic cells [30], [62] and the Clec4e receptor, and a consequent up-regulation of proinflammatory cytokines [34], [35]. The three pathways indicated above are all operational in up-regulation of MMP2, and to a lesser extent MMP9, and may actually be co-operative in lupus nephritis. Thus, it may be true that MMPs represent the factor that disintegrate membranes in progressive lupus nephritis and thereby provide access in situ for large chromatin fragments that have escaped DNaseI-mediated fragmentation.

A more detailed pathophysiological picture of lupus nephritis appears from data described here, and previously [5]. The process resulting in fulminant lupus nephritis may be summarized as follows. Appearance of anti-dsDNA antibodies, followed by formation of small chromatin fragment-IgG complexes and their deposition in the mesangial matrix represent the factors that induce mild or silent mesangial nephritis [5], [13]. The inflammatory milieu created by the early mesangial nephritis process may, although it deceptively appears as a clinically non-significant disorder, be the factors that silence the DNaseI gene expression. This model may be valid both for focal nephritis, and for global end-stage nephritis, depending on whether DNaseI is silenced globally in the kidney or only in smaller inflammation affected renal foci. The latter is currently under investigation in our laboratory. Thus, data presented here may point at early, clinically silent or mild mesangial nephritis as an inducer of DNaseI silencing. This indicates a wider effect on the genomic region and further epigenetic analyses are needed to explain this latter phenomenon.

Contemporary studies are consequently focused to identify the epigenetical mechanisms that account for silencing of the DNaseI gene. We are currently analysing two mechanisms; *i.* the role of regulatory RNAs, and have yet identified at least one microRNA in murine lupus nephritis that theoretically target DNaseI mRNA; and *ii.* transcriptional interference with closely situated genes. Of importance is also to identify the timely relationship between inducers of DNaseI gene silencing, and if the silencing is the direct cause for progression of lupus nephritis from mild mesangial nephritis into end-stage organ disease. This process may be relevant to understand the basis for focal as well as global lupus nephritis. In renal regions where DNaseI activity is low, un-fragmented chromatin may be retained and bound to membranes and targeted by relevant potentially nephritogenic anti-chromatin autoantibodies. Thus, focal lupus nephritis (see [63] for classification of lupus nephritis) may reflect focal glomerular exposure of chromatin fragments that have escaped degradation in regions with silenced DNaseI gene. If silencing of DNaseI appears globally in the kidneys of a given patient, this will promote a uniform end-stage disease.

To understand how the DNaseI gene is down-regulated in the kidney may bring us a significant step towards the understanding of the molecular and genetical events that in the end result in progressive lupus nephritis. This insight may also be important to develop new and causal treatment strategies, like inhibiting DNaseI gene silencing, or to inhibit binding of chromatin to membranes along strategies recently published [64].

## Supporting Information

Table S1 Complete sets of data from the RT2 ProfilerTM PCR Array mouse TLR signalling Pathway array plate (PAMM-018) is presented. Data represent average of gene expression levels in 3 parallel mice given as fold change (+SD) in Group1–3 mice. Data in Group 2 and Group 3 mice were normalized against data in Group 1 mice (set to 1). Relative expression levels were calculated using ddCT method. Selected sets of data from these array analyses are given in Table 1.
(XLS)
